# Quarantine Convention, Philadelphia, May, 1857

**Published:** 1857-07

**Authors:** 


### EDITORIAL DEPARTMENT.

---

*Quarantine Convention, Philadelphia, May*, 1857.—The absolute value of quarantine laws and regulations is among the most important of the questions presented to the sanitarian for his investigation and decision, while pursuring those inquiries which have for their object the prevention of diseases, especially such as assume the character of an epidemic or contagious type.

That a great variety of opinions exists upon the various questions involved in these considerations cannot have escaped notice. The vagaries of epidemics are at times such as to excite suspicion in the most cautious, of the utter inefficiency of any *cordon sanitaire* to limit the spread of disease; while on the other hand the close connections observable between cause and effect are too apparent to escape the observation of the most careless. Public opinion undoubtedly preponderates toward the side of a belief in the value of quarantine regulations in protecting the health of an exposed community. The instinct of preservation, intuitively prompts us to close the doors of entrance against the approaching foe, and though he should scale the housetop, and find some other entrance, the consciousness of having employed such measures of precaution as reason dictates, quiets all self-reproach and reconciles the sufferers to their fate.

There is, however, in the application of these measures of caution, undoubtedly a great want of a proper knowledge of the true principles upon which they should be applied. The arbitrary application of some of the existing laws may undoubtedly work to the injury of a community, financially at least, if not upon the score of health. And suspected persons, may be unjustly punished for the mere suspicion of a contagion lurking in their garments, by an incarceration in quarantine grounds. The value of these measures of precaution furnishes but a poor excuse for their misapplication and the injuries growing out of the same.

It would be a vain boast for science to pretend that it has fathomed all the depths of the mysteries concerned in the development and propagation of contagious and epidemic diseases. We at best, stand upon the very threshold of the subject, and the torch of science illumes as yet but a very short distance the deep darkness in which these subjects are hidden. Some advance has, however, been made, and whatever may be the sum of attainments, humanity calls for its application in its behalf.

An effort has at last been made to reduce to practical application what is known upon the subject, and to establish as far as practicable, a uniformity in the laws governing the entrance to our seaports. A convention of those, who from their official position, or scientific attainments have an interest in this subject, met in Philadelphia, on Wednesday the 13th of May last, and spent three days in the discussion of the numerous questions involved in the subject of quarantine.

This convention resulted in consequence of action recommendatory of such a gathering, being taken by the Board of Health, of Philadelphia, in the fall of 1856. The suggestion itself first was made by Dr. Wilson Jewell, a member of the Board of Health of that city. It resulted in soliciting the representation of the various official health departments in the seaport towns in a convention at Philadelphia. The proceedings of this convention are before us in a neat pamphlet of sixty pages. Although the delegations to the assembly was strictly confined to the seaport towns and cities, and to where quarantine laws are enforced, the subject is one of sufficient interest to claim the attention of the profession generally.

The convention was permanently organized on Wednesday, May the 13th, by the election of Dr. W. Jewell, President. Seventy-three delegates from nine states, and representing twenty-six authorities, were in attendance. Representatives were present from the Boards of Health of Boston, Providence, New York, Newark, Camden, Philadelphia, Wilmington, Baltimore, Norfolk, New Orleans; besides from several medical associations. The delegation was not strictly confined to the medical profession, and the lay members of the several Boards of Health freely participated in the discussions and business of the session.

During the temporary organization of the convention, and previous to the election of permanent officers, Dr. Jewell delivered an address, minutely detailing all the steps taken to bring the present representation together in convention; and also gave an outline of the quarantine regulations of some of the principal cities of the Union: the whole forming an intelligent basis upon which to predicate future action.

A committee of seven was appointed to prepare and arrange business for the convention. The next morning, Thursday, the committee presented a report, which was taken up by the convention for discussion, and each proposition made the subject of especial examination. This labor was completed on Friday, and resulted in the adoption of the following

### REPORT.

"*Whereas* great interest has been awakened in the questions pertaining to commercial intercourse among the nations of the earth, and to the close relations, under some circumstances, of the health of communities to the regulations which affect this intercommunication; and inasmuch as there is great diversity and irregularity in the rigor of enactment which characterizes the legislation of different bodies upon this subject; therefore be it

"*Resolved*, That it is expedient the system of quarantine regulations be revised, and that correct principles, as far as scientific research and observation have developed them, should be the basis of future enactments, to the end, that a uniform code, as far as practicable, may be secured in all our ports.

"*Resolved*, That the following propositions be regarded as the sentiment of this convention:

"1. There are certain diseases which may be introduced into a community by foul vessels and cargoes, and diseased crews and passengers.

"2. These diseases are small-pox, and, under certain circumstances, typhus fever, cholera, and yellow fever.

"3. When the latter diseases are introduced in this manner, their action is limited to individuals coming within their immediate influence, and cannot become epidemic, unless there exist in the community the circumstances which are calculated to produce such disease independent of the importation.

"4. The circumstances alluded to, consist in vitiated states of the atmosphere, from local causes, in connection with peculiar meteorological conditions.

"5. Efficient sanitary measures, including quarantine, will, in most cases, prevent the introduction of these diseases, and may, at any rate, disarm them of their virulence, and prevent their extension, when introduced.

"6. The present quarantine regulations, in operation in most of our States, are inefficient, and often prejudicial to the interests of the community.

"7. Diseases may be introduced: 1st, by a foul vessel, especially when proper measures are not taken to keep the hold free from stagnant and putrid bilge-water; and more particularly when there exist in the hold droppings or drainage from putrescible matters, which are allowed to penetrate and remain between the timbers of the ship. 2d. By cargoes, consisting, in whole or in part, of rags, cotton, or like porous substances, shipped from ports at which any malignant epidemic or endemic disease, of a contagious or infectious character, prevailed at the time when the vessel was loaded. 3d. By filthy bedding, baggage and clothing of emigrant passengers, particularly when these are crowded together in insufficient quarters, although the passengers themselves may be free from any actual disease. 4th. By the air that has been confined during a voyage in closely sealed or ill-ventilated holds. 5th. By squalid and diseased passengers landed and crowded together in unhealthy neighborhoods, or in small and ill-ventilated dwellings. 6th. By passengers and crews who are actually laboring under, or infected with any positively contagious disease, and their bedding, clothing and baggage.

"8. To prevent, therefore, the introduction of disease from the several causes enumerated, the necessity is apparent of providing a system by which all parts of a vessel may be ventilated during a voyage; and for the careful inspection of all vessels immediately upon their arrival, and before they are allowed to come up to the wharves of a city, for the landing of their passengers or the discharge of their cargoes. No vessel arriving between the 1st of May and the 1st of November, should, in fact, be admitted to a port until her hold is freely and fully ventilated, nor until the bilge-water is entirely r.moved.

"9. Provision should be made for the immediate landing of all those portions of the cargo of a vessel, and the baggage and clothing, that may be judged capable of generating or communicating disease; and for their proper purification, at such places and under such regulations as shall preclude all danger of their exerting a morbific influence, either immediately, or upon their subsequent admission into the city.

"10. Provision should be made for the immediate landing from on board of vessels as they arrive, of all persons who are actually laboring under disease, and for their due and comfortable accommodation and treatment, until such time as they can be taken charge cf, and properly cared for by their friends.

"11. In the case of a ship load of squalid passengers, or those strongly predisposed to disease, their clothing, beds, and other effects, should be at once subjected to a thorough ventilation and purification; and, upon their their landing, adequate measures should be adopted to prevent them from crowding together in confined, unhealthy, and ill-ventilated dwellings and localities.

"12. When a vessel arrives in a foul condition, or on board of which disease has prevailed during the voyage, after her crew and passengers have been removed from her, she should be subjected to a thorough process of cleansing and purification; for which purpose it may be necessary to discharge her cargo at a safe distance from the city, and to allow only such portions of it to be conveyed there as are incapable of creating disease, the residue being subjected to ventilation in such a manner as shall prevent it from suffering damage and all avoidable deterioration.

"13. The carrying out of these provisions should be intrusted to a single officer, with such assistants as may be required to aid him in the performance of his functions.

"14. This officer should be a regular physician, of unquestionable talents and experience, and possessed of great decision and rectitude of character.

"15. His compensation should be sufficiently ample to enable him to devote his entire attention and energies, throughout the year, to the duties of his office.

"16. While the power of removing him for incompetency, neglect, or other adequate cause, should be vested in some competent tribunal, his appointment should be based solely upon his capacity to fulfil satisfactorily his incumbent duties, and his continuance in office made dependent upon his faithful and skillful discharge of those duties.

"17. To this officer should be intrusted the sole and entire decision, under certain general provisions established by law, as to the treatment required in the case of each vessel that shall arrive, and of its cargo, crew and passengers, and to place it and these in a condition to prevent any danger of the introduction, by them, of disease; he, at the same time, being held to a strict accountability for the manner in which the discretionary power thus confided to him, is executed.

"18. As in every community a Board of Health is necessary to watch over its sanitary condition, and to prevent or remove all domestic sources of disease, this body would appear to be the one in which the power of appointing, and the general supervision of the official conduct of the Quarantine Physician may, with the greatest propriety, be invested.

"19. With a view to procure a uniformity in quarantine regulations throughout the several ports of the United States, the assembling of another, and probably several, conventions similar to the present one, will be required.

"20. To provide for the assembling of such a Convention in 1858, it is suggested that the President, Vice-presidents, and Secretaries of this Convention, with a committee of one member from each State represented in the Convention, be continued after our adjournment, as commissioners for the purpose of taking the necessary steps for the call of a Convention next year: *Provided, however*, that their powers shall cease immediately upon the assembling and organization of the Convention of 1858.

"21. A thorough examination should be made of all immigrants on their arrival; and if they are not protected against small-pox, they should be vaccinated.

"22. We recommend that there should be attached to our Boards of Health and Quarantine establishments, stations for minute meteorological observations, and vaccine establishments; and that records of these be published at stated periods for the public benefit.

"23. We advise the introduction of increased comforts for crews and passengers, and the ventilation and purification of vessels by a more effectual method."

The vote was taken by delegations, eighteen answered Yea, two answered Nay, and two reported a tie. The delegations from Massachusetts, Rhode Island, New York, New Jersey, Pennsylvania, Delaware, Maryland, voted in the affirmative. Virginia voted in the negative, and the two delegations from New Orleans each reported a tie vote.

It was resolved, that hereafter that the name of this Convention shall be The Quarantine and Sanitary Convention.

It was

"*Resolved*, That each municipal body represented in this Convention be recommended tc appoint one or more capable persons to keep a record of the invasion or origin and progress of future epidemics that may from time to time visit them, and return a copy of the same to this Convention.

It was

"*Resolved*—1. That in addition to the usual quarantine establishments, this Convention recommends the introduction of efficient means for removing all persons of limited means from infection, and for preventing the ingress of immigrants and other unseasoned people into ports and cities laboring at the time under pestilental diseases.

"2. That in all cases where rumors and unauthorized reports indicate certain ports or cities as the seats of epidemic and pestilential diseases of the nature provided against, no such reports shall be the basis for action elsewhere, unless sustained by an official declaration of the Boards of Health, or other properly constituted authorities.

"3. That all such Boards of Health and other public authorities, shall be obligated to declare the existence of invasions of yellow fever and cholera, in their epidemic forms, as they may from time to time make their appearance in the localities under their control.

It was

"*Resolved* That we recommend the adoption of a complete, accurate and uniform system of registration of births and deaths in all our cities, as a necessary accompaniment of efficient sanitary measures."

Baltimore was recommended as the next place of meeting of the convention, and it adjourned subject to the call of the President. J. M. N.

